# Efficacy of a text messaging (SMS) based intervention for adults with hypertension: protocol for the StAR (SMS Text-message Adherence suppoRt trial) randomised controlled trial

**DOI:** 10.1186/1471-2458-14-28

**Published:** 2014-01-11

**Authors:** Kirsty Bobrow, Thomas Brennan, David Springer, Naomi S Levitt, Brian Rayner, Mosedi Namane, Ly-Mee Yu, Lionel Tarassenko, Andrew Farmer

**Affiliations:** 1 University of Oxford, Institute of Biomedical Engineering, Oxford, UK; 2 Nuffield Department of Primary Care Health Sciences, University of Oxford, 23-38 Hythe Bridge Street, Oxford OX1 2ET, UK; 3 Chronic Disease Initiative for Africa, Division of Diabetes and Endocrinology, Department of Medicine, University of Cape Town and Groote Schuur Hospital, Cape Town, South Africa; 4 Division of Nephrology and Hypertension, Department of Medicine, University of Cape Town and Groote Schuur Hospital, Cape Town, South Africa; 5 Metro District Health Services, Cape Town, Western Cape, South Africa

## Abstract

**Background:**

Interventions to support people with hypertension in attending clinics and taking their medication have potential to improve outcomes, but delivery on a wide scale and at low cost is challenging. Some trials evaluating clinical interventions using short message service (SMS) text-messaging systems have shown important outcomes, although evidence is limited. We have developed a novel SMS system integrated with clinical care for use by people with hypertension in a low-resource setting. We aim to test the efficacy of the system in improving blood pressure control and treatment adherence compared to usual care.

**Methods/design:**

The SMS Text-message Adherence suppoRt trial (StAR) is a pragmatic individually randomised three-arm parallel group trial in adults treated for hypertension at a single primary care centre in Cape Town, South Africa. The intervention is a structured programme of clinic appointment, medication pick-up reminders, medication adherence support and hypertension-related education delivered remotely using an automated system with either informational or interactive SMS text-messages. Usual care is supplemented by infrequent non-hypertension related SMS text-messages. Participants are 1:1:1 individually randomised, to usual care or to one of the two active interventions using minimisation to dynamically adjust for gender, age, baseline systolic blood pressure, years with hypertension, and previous clinic attendance. The primary outcome is the change in mean systolic blood pressure at 12-month follow-up from baseline measured with research staff blinded to trial allocation. Secondary outcomes include the proportion of patients with 80% or more of days medication available, proportion of participants achieving a systolic blood pressure less than 140 mmHg and a diastolic blood pressure less than 90 mmHg, hospital admissions, health status, retention in clinical care, satisfaction with treatment and care, and patient related quality of life. Anonymised demographic data are collected on non-participants.

**Discussion:**

The StAR trial uses a novel, low cost system based on widely available mobile phone technology to deliver the SMS-based intervention, manage communication with patients, and measure clinically relevant outcomes. The results will inform implementation and wider use of mobile phone based interventions for health care delivery in a low-resource setting.

**Trial registration:**

NCT02019823

## Background

Globally, high blood pressure is the leading single risk factor for cardiovascular and related diseases [1,2]. Individual patient-level data have shown that usual blood pressure is “strongly and directly” associated with cardiovascular and overall mortality risk [3], and that lowering blood pressure reduces this risk [4-6]. Controlling blood pressure at a population level may be associated with a decrease in cardiovascular disease regardless of the increasing burden of obesity and diabetes [7]. The burden of disease associated with hypertension is greatest in low-resource settings [2,8], where there is a need for better control of blood pressure [9,10].

Treatment to lower blood pressure includes drug and non-drug measures as well as modification of known life-style associated risk factors, such as smoking and alcohol intake [6,11,12]. Long-term blood pressure control requires integrating medication taking into daily life to support adherence and persisting with treatment.

A wide range of different strategies and interventions has been used to support patients in adhering to treatment plans, although results are not consistent [13]. One strategy that may facilitate such support is the integration of a patient’s mobile (or cellular) phone in the process of health care delivery. Globally, the mobile phone has become the preferred tool for communication and access to information bringing together communication and computing technology. Mobile-phone-based interventions could address individual-level factors in health by facilitating timely patient access to relevant health information and support, making patient-provider communication easier, and providing context-specific support and prompts to action [14].

Despite widespread interest in the potential for mobile phone based interventions to deliver disease management and health-related behaviour change sustainably and at scale, published evidence of effectiveness is limited [15]. SMS-text messages are available on all mobile phone platforms and with all providers. Text messages have the potential to provide information alone (informational) or provide two-way communication (interactive or bi-directional). Some but not all trials suggest that SMS text-message reminders may improve clinic attendance, retention in care, and self-reported medication adherence [15]. Two trials in Spain with short follow-up periods have explored the efficacy of SMS-based interventions for people with hypertension to improve treatment compliance and blood pressure control. Although the trials results suggest potential for small to moderate effect sizes in improved compliance and blood pressure control, the effects were not statistically significant [16].

## Methods

### Design and hypothesis

**Figure 1:**
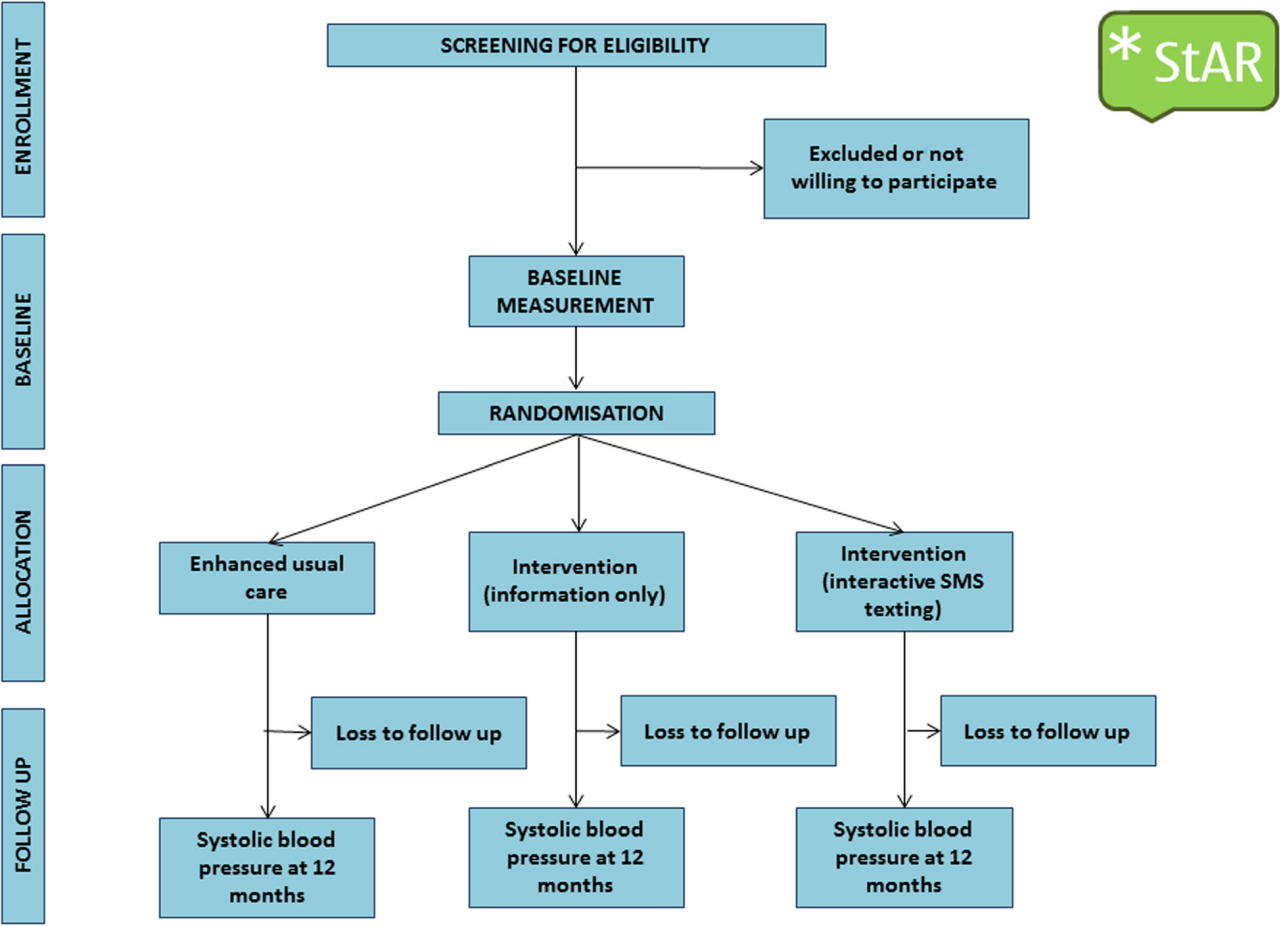
CONSORT diagram for SMS-text Adherence suppoRt Trial (StAR).

The SMS Text-message Adherence suppoRt trial (StAR) is a twelve-month, pragmatic three-arm parallel group trial. Participants are individually randomised to one of two trial intervention groups or an enhanced usual care group (control) in a 1:1:1 allocation ratio (Figure 1).

We therefore plan to test whether a structured intervention delivered by an automated system of SMS text-messages providing clinic appointment and medication pick-up reminders, medication adherence support and hypertension-related education delivered remotely through informational or interactive SMS text-messages is more effective than usual care in controlling blood pressure.

### Setting

Participants are recruited from the Vanguard Community Health Centre, a public sector, large primary care clinic in Bonteheuwel, Cape Town, South Africa, serving two diverse low-middle income communities, Langa and Bonteheuwel. The health centre provides a full range of primary care services with approximately 28,000 people attending each month. The “chronic diseases of lifestyle clinic” services a caseload of about 500 patients per week. Patients with hypertension are managed by nurse practitioners and doctors in structured clinics. The local Department of Health provides medications for the treatment of hypertension in line with national and international hypertension management guidelines [17]. Medications are provided either pre-packed from an off-site pharmacy, or dispensed on-site. Medications are usually provided for 28 days. Patients are required to attend the clinic monthly to collect their medication. There is no fee for receiving care or medication in primary care.

### Participants

Trial eligibility criteria are: (i) a clinical diagnosis of hypertension and either currently receiving blood pressure lowering medication or are about to start such medication, (ii) a systolic blood pressure <220 mmHg and a diastolic blood pressure <120 mmHg at enrolment (iii) aged 21 years or older, (iv) own or have daily access to a cell-phone, (v) able to send a SMS text-message, or can do so with help of a relative, (vi) do not live in a household where another member has been recruited into the trial, (vii) not pregnant or within three months post-partum by self-report, (viii) currently residing in the trial area and expecting to be resident for the duration of the trial and (ix) willing to give informed consent to take part in the trial. Patients with a systolic blood pressure >175 mmHg or a diastolic blood pressure >105 mmHg are eligible only if they have no symptoms suggesting a hypertensive emergency and they have been evaluated by a medical officer.

### Interventions

The two active interventions are delivered by an automated system of SMS text-messages providing clinic appointment and medication pick-up reminders, medication adherence support and hypertension-related education delivered remotely using either informational or interactive SMS text-message.

#### Intervention development

**Figure 2:**
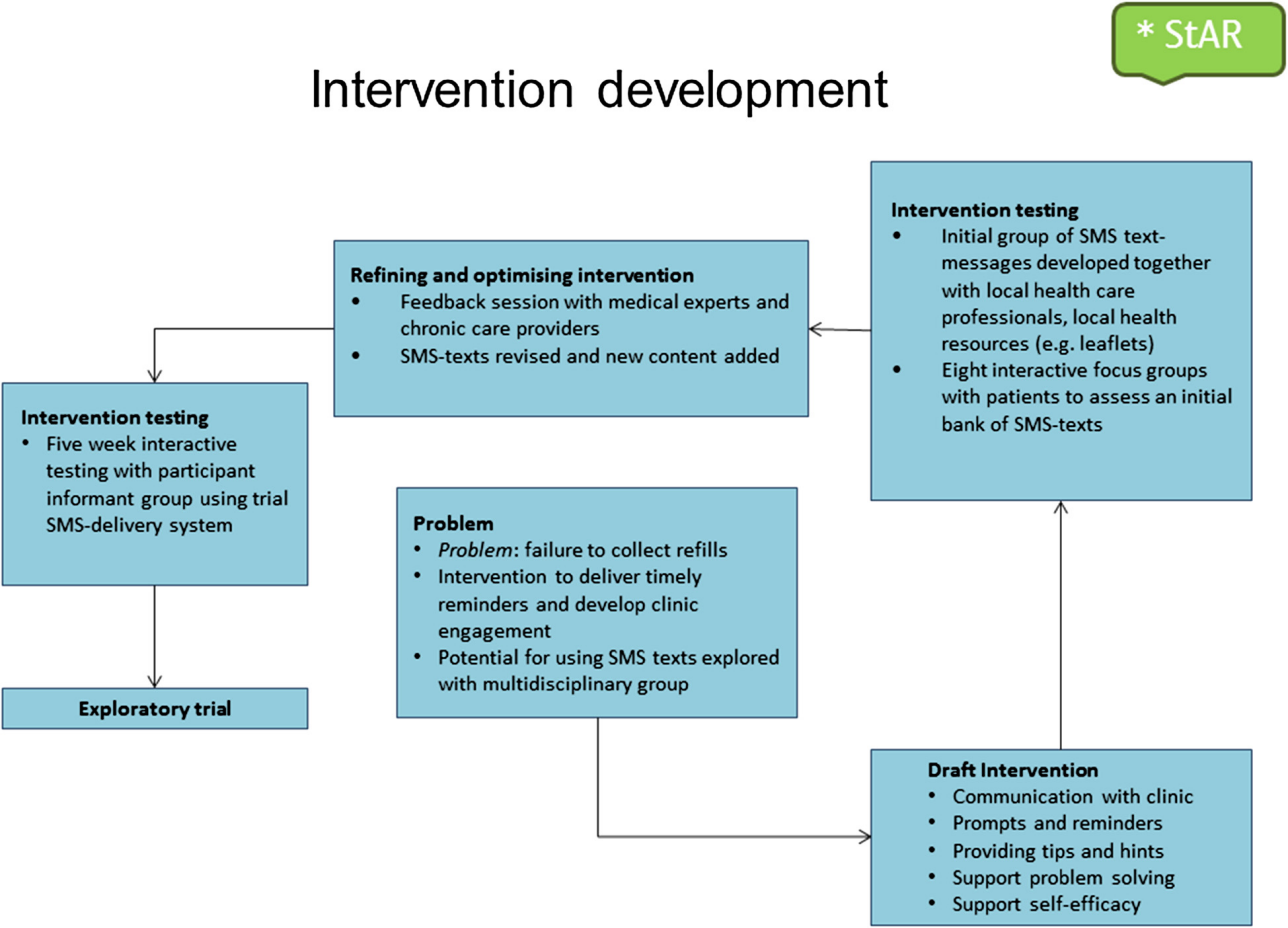
**Process of intervention development adapted from Smith et al. [**[18]**].**

We iteratively designed, developed, and tested the interventions using a formal process to synthesise existing evidence, the opinion of clinical staff, and patients with chronic diseases (Figure 2). [18,19]As part of this process we developed a bank of SMS-text messages which we mapped on a taxonomy of behaviour change techniques. [20] Most of the content focused on the techniques of goals and planning, repetition and substitution, social support, and natural consequences (Table 1). The SMS text-messages used in the intervention were developed, translated, and tested in English, Afrikaans and isiXhosa, the three languages most commonly spoken by people living in Cape Town.

**Table 1:** Examples of trial text messages mapped to behavioural change taxonomy


Goals & planning	A pill box can help you to remember when to take you high blood pills. We encourage you to get one. For more info ask PHARMACY.
	Make taking your pills part of your daily routine, like when you brush your teeth. Doing this can help you remember to take them regularly.
Repetition & substitution	Pls remember your next CLUB DATE is on [DAY][DD/MM/YY] at [TIME].
	Thnx for picking up your meds. Keeping on your pills & attending on your correct dates helps us serve you better.
	We missed you @ CLUB. We hope you’re OK. Pls be sure to come in to collect your MEDICINE.
Social support	Ask someone you trust to help you remember to take your medicine as directed.
	You’re doing very well. Pls keep on with your pills, come on your clinic dates, exercise&eat healthy food.
Natural consequences	Pls remember your high blood can’t be cured. To keep healthy pls keep on with your pills, come on your clinic dates, exercise&eat healthy food.

#### Informational (unidirectional) SMS text-message system

Participants allocated to the informational SMS text-message group receive an intervention that includes reminders about medication collection, scheduled clinic appointments and follow-up text-messages (including reminders when medicines are not collected), and a SMS-text promoting specific adherence-related behaviours. The SMS-texts are sent weekly at a time and in a language selected by the participant. Some of the texts are personalised (include first or chosen name). The intervention is compliant with South African regulations and ethics, and regulatory guidelines on good SMS-practice [21].

#### Interactive (bidirectional SMS text-message reminders)

In addition to the intervention provided to participants allocated to the informational SMS text-message group, participants in the interactive SMS text-message group can also respond to texts and send messages to the clinic. For example, when appointment reminders are sent to participants in the interactive SMS text-message group, they will include an option to reply if they cannot make their scheduled appointment and to cancel or change their appointment as necessary. Examples of the type of SMS text-messages sent to participants allocated to the two intervention arms are shown in Table 2.

**Table 2:** Examples of intervention group SMS-text messages in English


• **Appointment reminder SMS-texts**	*Hi [insert name]. Pls remember your next MEDICINE DATE is on [DAY][DD/MM/YY] at [TIME]. Thnx Dr [NAME] (DR@VAN)*
• **In response to missing a scheduled appointment**	*We missed you @ PHARMACY. We hope you’re OK. Pls be sure to come in to collect your MEDICINE. Thnx Dr [NAME] (DR@VAN)*
• **Messages related to specific issues**	*A pill box can help you to remember when to take your high blood pills. Ask at PHARMACY where you can buy one. Thnx Dr[NAME](PHARM@VAN)*
	*Pls be sure to take all your medicine as directed even if you feel well. Thnx Sr [NAME](SR@VAN)*
	*Pls tell us(DR&PHARMACY)if you think your high blood pills are making you feel unwell. Ask us about common side effects of your pills. Thnx Dr [NAME](DR&PHARM@VAN)*

#### Usual care

Participants allocated to the usual care group only receive the SMS-texts sent to all trial participants. These messages are sent no more frequently than one SMS text-message every four weeks. The messages are a welcome SMS text-message, a text confirming enrolment, an SMS text-message on a birthday, and other SMS text-messages about participation in the trial. Examples of the types of SMS texts-messages sent to all trial participants can be seen in Table 3.

**Table 3:** Examples of trial-specific group-text messages in English


**• Welcome SMS-text message**	Welcome to StAR* thank you for participating. The trial cell is 0XXXXXX120 (save on phone). We will never ask for personal or banking details. Dr[NAME](StAR*@VAN)
**• Trial specific SMS-texts**	Pls remember your participation in StAR* is voluntary. Call, text, or send plz cal 0XXXXXX372 (its free) if you need to withdraw. Thnx Dr [NAME] (StAR*@VAN)
	*Pls remember participating in StAR is free. We do not charge you for the SMS-texts we send to you. Thnx Dr [NAME] (StAR@VAN)*
**• Birthday SMS-text**	Hi [NAME]. Happy birthday, have a great day. Thnx [NAMES](Health Team & StAR*@VAN)

### Procedures to promote intervention fidelity and reduce between-group contamination

Clinic staff and trial participants are told that the trial procedures involve collecting information about the number, timing, and content of text-messages for health and thus not everyone will be receiving the exact same number or type of messages. Participants are asked not to share the SMS text-messages with others. SMS text-message content is stored remotely in a message bank on a secure server. An automated delivery system is used to send SMS text-messages and to manage interaction with trial participants at no cost to participants (Table 4). The server and delivery system are managed by technicians with no direct contact with trial participants. Trial staff are clearly identifiable and distinct from clinic staff. A trial log is kept to record and describe any unscheduled contact between trial participants and the trial team.

**Table 4:** Practical aspects of sending and receiving SMS text messages


**SMS text message**	• Receiving SMS text messages or phone calls is free in South Africa
	• SMS text messages can be sent to participants through any of the local mobile phone network providers
**“Please Call Me” (PCM)**	• PCM messages are equivalent to a missed-call and are freely available (a minimum of five PCMs are available daily) across all local networks
**Devices or credit**	• Participants are not be provided with mobile phones or network airtime credit

### Outcomes

#### Primary outcome measures

The primary clinical outcome is change in mean systolic blood pressure from baseline to twelve months measured with a validated electronic device (IEM Stabil-o-graph©) using a standard protocol [22]. We record six sequential blood pressure readings at 3-minute intervals. Data from the IEM Stabil-o-graph© are uploaded to trial database and the reported blood pressure is calculated by discarding the initial reading and calculating the mean from the five remaining readings. [23]

#### Secondary outcomes

Treatment adherence is assessed by calculating the proportion of days of medication covered from routinely collected prescribing and dispensing data obtained during the 12-month follow-up period [24]. Data will be reported in three-monthly intervals. Adherence will be reported as the proportion of patients with ≥80% of days covered with medication [25], and as a continuous measure.

Additional secondary outcomes are: proportion of participants achieving a systolic blood pressure less than 140 mmHg and a diastolic blood pressure less than 90 mmHg, and health status measured with the EuroQol 5-Dimension and Visual Analogue Scale measures [26], proportion of scheduled clinic appointments attended, retention in clinical care, satisfaction with clinic services and care using 12 five-point Likert-scale items adapted from locally appropriate questionnaires developed to capture patient satisfaction with services for HIV-care, [27,28] hospital admissions, a Visual Analogue Scale for self-reported adherence to medication, and an adapted four-point scale evaluating basic hypertension knowledge. [29] We are also collecting data on the number and type of medication changes made during the trial and reported side-effects of medication. Anonymised demographic data are collected on non-participants.

### Trial procedures

The trial specific procedures to recruit, enrol and follow-up participants are integrated within existing health care delivery pathways. Participants continue to receive clinical care from their usual care providers.

#### Baseline data collection

Participants are recruited from patients attending any of the chronic disease services at the health centre. Potential participants are given information (verbal and written) about the trial and, if willing, screened for eligibility. All trial information is available in English, isiXhosa, and Afrikaans. The trial screening protocol includes the standardised measurement of height, weight, waist circumference, and blood pressure using a validated electronic device and the data are entered into the patient’s clinic record. Eligible individuals interested in participating have the opportunity of asking trained research staff further questions about the trial and sufficient time to consider whether or not they wish to participate.

Consenting participants attend a baseline consultation of 20 minutes with a trained research assistant. After obtaining written confirmation of informed consent and detailed contact information including the participant’s mobile-phone number, the data are uploaded and the system sends a “Welcome” SMS-text message to the participant, allowing further discussion of the SMS-based support that will be available in the trial. Participants select the language, and the day and time of the SMS-messages they will receive. No further specific training is given in how to access or interpret the message. Confirmed receipt of the SMS-text (either in real-time or within 72 hours of registration) is a required for completion of the enrolment procedure and trial randomisation. The research assistant then administers a short baseline questionnaire; details of current clinical management and medication are extracted from paper-based and electronic records from the clinic and other Department of Health service providers.

#### Follow up to 12 months

Trial participants are followed upwhen they attend for planned clinic appointments. Data on scheduled clinic appointment attendance, prescription refill collection, and drug availability are extracted from routinely collected service-delivery data. At the six-month visit participants have their blood pressure measured and their contact details updated if necessary. At the final twelve month follow-up, anthropometry is repeated and blood pressure measured, a follow-up questionnaire is administered by a research assistant and clinical records are reviewed to capture intervening clinical events and changes in medical management. Participants who miss their scheduled visit at 12 months are actively traced by community-based research staff; if cause for non-attendance is hospital admission or death, then hospital records or death certificate data are obtained.

### Sample size calculations

The trial is powered on the primary outcome measure and based on a comparison of mean blood pressures between the usual care group and each of the intervention groups at 12 months.

A decrease in systolic blood pressure of 5 mm Hg is associated with clinically important reduction in the relative risk of stroke and coronary heart disease events. [4] Based on a study population similar to that expected for the StAR trial population we used the standard deviation (SD) of systolic blood pressure (22.0 mm Hg) to calculate the required sample size. [9]

With a proposed sample size of at least 1215 participants (no fewer than 405 for each group) and a 1:1:1 allocation ratio, the trial will have power of 90% with an alpha of 0.05 (two-sided), to detect an absolute mean difference of 5 mm Hg in the change in systolic blood pressure at 12 month from baseline, allowing for up to 20% loss to follow up.

### Randomisation

Following receipt of a “Welcome” SMS text-message confirming trial registration on the system, participants are randomised using a secure, remote, web-based computer schedule within one week of recruitment. A minimisation procedure, overseen by an independent statistician, is used to dynamically adjust randomisation probabilities minimising chance differences in gender (male, female), age (<55 years, ≥ 55 years), baseline systolic blood pressure (<140 mmHg, ≥ 140 mmHg), years with hypertension (< 10 years, ≥ 10 years), and previous clinic attendance (regular, irregular, unknown) between the groups. Research staff and clinic staff remain blind to the allocated treatment group.

### Data collection and management

We used software platforms that are non-proprietary, open-source, stable and widely available to deliver the mobile-phone based intervention at scale in a low-resource setting. We selected software that was easily customisable and suitable for use by non-technical field staff. Patient data is collected by fieldworkers using Internet-enabled mobile phones (HTC Wildfire S, HTC Corporation, Taiwan, Android OS 2.3.5) and tablet computers (Motorola Xoom, Motorola Corporation, Illinois, Android OS 4.0.4). Data entry is through on-screen case report forms developed using the open-source mobile-device application Sana Mobile (Sana, MIT, Massachusetts). The medical record is held in OpenMRS version 1.6.1 (OpenMRS, Limited, Michigan). OpenMRS is an open-source client–server software platform initially designed as an electronic medical record and health system management platform for low-resource settings. The database uses a “concept dictionary” which stores the terms describing each data variable. It is accessible through a web-based interface and the platform allows the development, upload and installation of custom-coded modules into the main framework enabling seamless interactivity with Sana Mobile. Participant blood pressure readings are transferred directly from the sphygmomanometer to the tablet computer using a Bluetooth™ protocol. Data are then securely transferred to a dedicated mobile dispatch server (MDS) using SSL transport layer encryption (data are uploaded incrementally to handle intermittent data connectivity). The MDS then compiles and uploads all the information to an individual patient electronic medical record in OpenMRS.

Data collected using Sana Mobile are error-checked using range and logic checks built into Sana at time of collection. All other data uploaded directly to the database, such as patient appointment attendance, are error-checked by the OpenMRS software before integration on the server. These include data format and range checks, appointment date checks and alignment between uploaded and existing data. The database is regularly backed up and password-protected, with differing levels of access for different research staff.

### Statistical methods

#### Analysis of primary and secondary outcomes

The primary statistical analysis will be carried out on an intention-to-treat (ITT) basis. We will endeavour to obtain full follow-up data on every participant to allow a full ITT analysis. The results from the trial will be reported as comparative summary statistics (difference in response rate or means) with 95% confidence intervals. All statistical tests will use a 5% two-sided significance level. The trial results will be reported in accordance with the CONSORT (Consolidated Standards of Reporting Trials) 2010 statements. A full detailed analysis plan (including plans for subgroup analysis and sensitivity analysis) will be prepared and finalised before unblinding of the data.

We will analyse the primary outcome (change in systolic blood pressure at 12 months from baseline) using a mixed-effects model on data collected at 6 and 12 months. An interaction between time and randomised group will be fitted to allow estimation of treatment effects (i.e. informational versus usual care, and interactive versus usual care) at each time point. The model will adjust for baseline systolic blood pressure and minimisation factors.

The secondary outcome measure of adherence will be dichotomised with a threshold of 80% (of days covered) to determine the proportion of participants judged adherent to medication. We will use chi-squared tests to examine differences between allocated groups.

#### Sub-group analyses

We will carry out sub-group analyses of the primary outcome, systolic blood pressure and the main secondary outcome, adherence over last three months, by age (<50 years, ≥50 years), sex (male, female), number of years with hypertension (<1, 1–5, >5), baseline blood pressure (>140, ≤140 mmHg), presence of one or more co-morbid conditions, and self-reported adherence at baseline.

Missing data will be reported with reasons given where available and the missing data pattern explored. We will explore the mechanism of missing data, though the mixed-effects model does implicitly account for data missing at random.

### Reporting of adverse events for this trial

Reporting of serious adverse events will be limited to events not already listed as primary or secondary outcomes, but which might reasonably occur as a consequence of the trial. Additionally we will record adverse events that might be reasonably related to SMS text-messaging including hand or finger pain, or involvement in an accident as a result of sending or receiving a text.

### Ethical approval

The Human Research Ethics committees of the University of Cape Town, the University of Oxford, and the Metro District Health Services, Western Cape have reviewed and approved the protocol (HREC UCT 418/211, HREC OXTREC 03–12). Trial procedures are compliant with the Declaration of Helsinki and the trial is registered with the South African National Clinical Trials Register (SANCTR DOH-27-1212-386) and ClinicalTrials.gov (http://NCT02019823).

### Consent

Eligible, willing participants complete a written consent form during the initial trial visit. Any participant who has given consent but loses capacity to consent during the trial will be withdrawn. Identifiable data already collected with consent will be retained and used in the analysis.

### Publication and dissemination of results

The results of the trial will be published in a peer-reviewed journal and will be available on PubMed Central within six months of publication. Dissemination of results to patients will take place via community meetings, patient organisations, and the local media. Trial data will be made available on a public access database.

## Discussion

This protocol presents the design of a randomised controlled trial testing the efficacy of a text-messaging intervention to support patients in their treatment adherence and improve blood pressure control. This is the first controlled trial testing the efficacy of text messaging in a large population for hypertension, at low cost and in a low-resource setting. The technology support for this trial is innovative, based on open-source software, and not only supports an interactive messaging system using standard and widely available SMS messaging, but also integrates clinical care and trial management.

In addition to testing the efficacy of the intervention, this study will provide information to further develop clinical care in low-resource settings and maximise the usefulness of new technology at low cost. It will provide an evaluation of the feasibility and acceptability of integrating different aspects of clinical care through a delivery platform which uses the patient’s mobile phone as an identifier and means of direct contact. The setting provides a particular challenge, set in a large peri-urban public sector clinic with a high patient load, typical of urban centres in Africa and the global south. We are recruiting participants from a clinic facility that are likely to be broadly representative of those receiving care for hypertension at primary care level.

The trial has been designed to address the limitations of previous studies of SMS text-messages to support treatment adherence for chronic diseases; [30] the intervention was developed using a formal process to synthesise existing evidence, the opinion of clinical staff, and patients with chronic diseases, and iteratively tested in pilot work. There is a large bank of possible messages, and the delivery system (including interactive bi-directional SMS text-messaging) is automated. The duration of the intervention and the length of follow-up will allow us to explore effects of the intervention on adherence and persistence with treatment, and to assess whether habituation to the intervention might moderate the effect. The outcomes include measurement of adherence, as a key explanatory variable associated with improved cardiovascular outcomes, and blood pressure as a clinically relevant outcome.

## Competing interests

The authors declare that they have no competing interests.

## Authors’ contributions

KB, TB, NL, BR, MN, LT, AF conceived of the study. All authors participated in the design and coordination of the study. LY, AF, KB, prepared statistical analysis plan. All authors read and approved the final manuscript. The contributions of other members of the StAR study team are gratefully acknowledged and listed below.

## Pre-publication history

The pre-publication history for this paper can be accessed here:

http://www.biomedcentral.com/1471-2458/14/28/prepub

## The StAR Team

Trial steering committee: Carl Lombard (Chair), Lionel Tarassenko, Karen Sliwa, Bob Mash, Tracey Naledi; Naomi Levitt and Andrew Farmer.

Trial management committee: Andrew Farmer, Naomi Levitt, Brian Rayner, Krisela Steyn, Kirsty Bobrow.

Coordinating Centre Oxford: Institute for Biomedical Engineering (IMBE) staff, Primary Care Trials Unit (PCTU) staff.

Coordinating Centre Cape Town: Carmen Delport, Carmelita Sylvester, Susan Botha, Chantel Stuart.

Intervention Development: Thomas Brennan, Nomazizi Cishe, Ntobeko N’wagi, David Springer.

Trial research assistants: Liezel Fisher, Melissa Paulse, Nicolette Gertse, Theresa Louw, Ntombi Ngabom, Nomfuneko Konzapi, Desmond Raqa, Nombulelo Ralarala, Luyanda Sebe.

Vanguard clinic staff: Mosedi Namane, Luntu Mbanga.
